# Operation for Radical Cure of Umbilical Hernia in a Colt Ten Months Old

**Published:** 1895-02

**Authors:** A. W. Clement


### OPERATION FOR RADICAL CURE OF UMBILICAL HERNIA IN A COLT TEN MONTHS OLD.

BY A. W. CLEMENT, V.S.

A FASHIONABLY bred "Standard" colt developed a hernia while at the farm on which he was bred in another State. This fall the colt and mare were sent to the owner, a client of mine, near Baltimore, and I was called in to prescribe for the animal. I found an umbilical hernia as large as a goose-egg, which I was unable to reduce while the animal was standing. On manipulation, moreover, it appeared that the ring was closing in on the loop of intestine, and that an adhesion had formed between the intestine and the edges of the umbilical ring.

The animal was cast, secured, and placed upon his back. Still it was impossible to reduce the hernia. I gave it as my opinion that the only method of reducing the hernia would be to chloroform the animal; and perhaps when the muscles were relaxed that reduction might be possible without cutting, in which case the application of clamps to the loose fold of skin opposite the opening would effect a cure. In case reduction were impossible, then it would be necessary to divide the ring, break down the adhesion, and sew up the edges of the ring, then amputate the loose fold of skin and stitch up the external wound.

The owner decided to leave the whole matter in my hands. It was determined to operate as soon as possible. Some few days afterward the animal was prepared for the operation. He was cast by the side lines, both front feet tied together and controlled by a rope in the hands of an assistant. Chloroform was administered slowly, and when well anæsthetized ether was used for the operation.

After placing the colt on his back, with his rump elevated about eight inches higher than his shoulders, the hair was shaved over the hernia and for some distance around it, and the skin disinfected as well as possible by permanganate of potash and oxalic acid. An incision was then made through the skin at the base of the hernia and extended to the point, then through the subcutaneous tissue and through the cyst-wall, exposing the intestine. It was then apparently impossible to push the intestine through the ring. With a curved bistoury the ring was enlarged so as to admit the little finger. It was then found that there was no adhesion at the ring. Further exploring and exposure of the loop demonstrated the adhesion at the point of the hernia. This adhesion was comparatively easily broken down by the finger, and the intestine then naturally returned to the cavity. The walls of the ring were brought together by four silver-wire sutures, tied but not twisted. The fibrous tissue forming the walls of the sac and extending directly from the walls of the ring was removed, some of the skin cut away, and for the posterior two-thirds the edges brought together by a continuous subcutaneous wire suture. Three interrupted wire sutures were used in the anterior somewhat dependent part of the wound, so that one or more could be removed, if necessary, to allow for drainage.

The operation was performed on the afternoon of January 21st. At 9 o'clock that evening the temperature was 100°; at 11 P.M., 102°; at 3 A.M., January 22d, 101°; at 9.30 A.M., 100°; at 6 P.M., 101.3°; and at 11 P.M., 102.3°. On January 23d, at 7.30 A.M., it was 103.3°. By this time there was a good deal of swelling and at the anterior part of the wound considerable fluctuation. An incision made through the skin allowed about an ounce of blood and pus to escape. The first suture was removed and good drainage obtained. At 6 P.M. the temperature was 102°, and by the evening of the 24th it was normal, and has remained so since. The discharge gradually became less, none of the stitches broke, the swelling gradually subsided, and in two weeks from the time the operation was performed the wound had entirely healed and the reduction of the hernia is now complete. A canvas bandage, with elastic straps on the sides, was made to fit the body, and laced on the back to hold the dressings and at the same time act as a support for the hernia. This bandage will be worn for a time to assist in the process of healing. The pulse was good and strong all of the time, never over 50, appetite good, but not allowed to eat much.

The mistake in the operative procedure was in not fully exposing the cyst before enlarging the ring. Had this been done the adhesion at the point would have been seen. This could have been broken down, when probably the intestine would have slipped back into the abdominal cavity without enlarging the ring. Probably this would have made the closing of the ring stronger.

